# Recent Advances in Tunable and Reconfigurable Metamaterials

**DOI:** 10.3390/mi9110560

**Published:** 2018-10-31

**Authors:** Sanghun Bang, Jeonghyun Kim, Gwanho Yoon, Takuo Tanaka, Junsuk Rho

**Affiliations:** 1 Department of Mechanical Engineering, Pohang University of Science and Technology (POSTECH), Pohang 37673, Korea; bsh9716@postech.ac.kr (S.B.); j941230@postech.ac.kr (J.K.); faofai@postech.ac.kr (G.Y.); 2 Metamaterials Laboratory, RIKEN Cluster for Pioneering Research, Saitama 351-0198, Japan; t-tanaka@riken.jp; 3 Innovative Photon Manipulation Research Team, RIKEN Center for Advanced Photonics, Saitama 351-0198, Japan; 4 School of Materials and Chemical Technology, Tokyo Institute of Technology, Tokyo 152-8550, Japan; 5 Department of Physics, Gakushuin University, Tokyo 171-8588, Japan; 6 Department of Chemical Engineering, Pohang University of Science and Technology (POSTECH), Pohang 37673, Korea; 7 National Institute of Nanomaterials Technology (NINT), Pohang 37673, Korea

**Keywords:** metasurface, perfect absorber, wavefront engineering, color filter, plasmonics, phase change material, graphene, indium tin oxide

## Abstract

Metamaterials are composed of nanostructures, called artificial atoms, which can give metamaterials extraordinary properties that cannot be found in natural materials. The nanostructures themselves and their arrangements determine the metamaterials’ properties. However, a conventional metamaterial has fixed properties in general, which limit their use. Thus, real-world applications of metamaterials require the development of tunability. This paper reviews studies that realized tunable and reconfigurable metamaterials that are categorized by the mechanisms that cause the change: inducing temperature changes, illuminating light, inducing mechanical deformation, and applying electromagnetic fields. We then provide the advantages and disadvantages of each mechanism and explain the results or effects of tuning. We also introduce studies that overcome the disadvantages or strengthen the advantages of each classified tunable metamaterial.

## 1. Introduction

A metamaterial (MM) is composed of nanostructures, which are called artificial atoms. These structures provide the MM with unique properties that natural materials cannot have. These properties can be used to overcome optical limits caused by several effects such as the diffraction limit, so, the use of MMs can yield abnormal properties in the devices. Super-lenses, hyper-lenses, and super-resolution imaging [1,2,3,4,5,6,7,8,9,10], negative index materials [11,12,13,14,15,16], invisibility cloaking [17,18,19], perfect absorbers [20,21,22,23], meta-holograms [24,25,26], artificial chirality [27,28,29], and electromagnetically-induced transparency [30] are examples of MM applications that have properties that do not occur in normal materials. However, before MMs can be used in practical ways, several challenges such as 3-dimensional MM fabrication [31,32] and fixed properties must be overcome.

The changeable properties of MMs have been achieved by introducing active materials such as vanadium dioxide (VO_2_), indium tin oxide (ITO), polydimethylsiloxane (PDMS), graphene, or liquid crystals. An active material provides an MM with tunability, reversibility, repeatability and fast response to change. We have classified tunable MMs into four categories according to the phenomenon that causes the change: thermal, optical, mechanical, and electrical effects. Some cases are ambiguous.

Thermal tunability uses temperature-responsive materials such as VO_2_, and germanium-antimony-telluride (Ge_2_Sb_2_Te_5_, GST). These are phase-changing materials in which the chemical structure (crystalline and amorphous) or material state (dielectric and metal) changes abruptly in response to temperature. These materials can be either used as a part of the layer or as the whole layer with additional processing. In addition, special polymers, distilled water, and liquid crystals have been used in thermally-tunable MMs. Typical advantages of these materials include a wide tuning range and the possibility to be used in integrated circuits; typical disadvantages are slow modulation and a tuning range that is not in the visible spectrum.

Optical tunability exploits materials that change in response to light. Examples include ITO, optical response polymers, and nanocomposite. The reflectance, resonance wavelength, and polarization of these materials can be controlled by adjusting the light or pumping power. Typical advantages of these materials include rapid response, tunability in the visible spectrum, and applicability in integrated circuits; typical disadvantages include a narrow tuning range and unfeasibility of controlling each cell.

Mechanical tunability exploits the dependence of an MM’s properties on their shapes or arrangements. This characteristic can be exploited by fabricating MMs on an elastic substrate, then stretching it, or by introducing microelectromechanical systems (MEMS) into MMs. The MEMS structure can induce a change in shape directly when a voltage is applied and can thereby tune the properties of the MMs. Physical deformation using piezoelectricity, magnetoelectricity, and the controlling strain field can also be used to tune MMs.

Electrical tunability exploits change in the electrical properties of materials. Many electrical properties are related to optical properties, so they can also be manipulated electrically. For example, if the number of electrical carriers changes efficiently according to the voltage, as in ITO, the optical properties of the materials may also change efficiently. Other methods include designing the MM two-dimensionally while controlling the number of carriers and using liquid crystals.

## 2. Thermally-Tunable Metamaterials

Many materials have a property that changes quickly with temperature; these have been exploited in MMs. Vanadium dioxide (VO_2_) and germanium-antimony-telluride (Ge_2_Sb_2_Te_5_, GST) are widespread materials for thermally tunable metamaterials. VO_2_ changes from a dielectric to a metal or vice versa at a transition temperature (68 °C) [33]. The thermal tuning of surface plasmon resonance has been achieved in Au-VO_2_ structure [34,35,36,37]. GST undergoes a phase transition between amorphous and crystalline phases; the transition is controlled by cooling speed. Rapid cooling from above melting temperature prohibits atoms from crystallization. As a result, it makes GST become amorphous; it can be changed to a crystalline phase by a series of heating processes between melting temperature and glass transition temperature [38]. 

Generally, the thermal tuning of MM has the advantages of a sufficient tuning range and compatibility of incorporation in integrated circuits [39]. However, it has the disadvantages of a slow modulation speed due to the time required for heating and cooling, and a tuning scope that is outside of the visible spectrum.

### 2.1. Thermally-Responsive Material: VO_2_

VO_2_ can be used as the whole layer on the substrate. The thermal tuning of localized surface plasmon resonance (LSPR) has been demonstrated experimentally using Ag nanoparticles on a thin film of VO_2_ [40]. The shifting range of the LSPR wavelength depends on the size and mass thickness of the Ag nanoparticles, and this shifting is reversible [41,42,43]. As the mass thickness increases, the surface plasmon resonance (SPR) wavelength increases due to the dipole-dipole interaction of the particles. The simultaneous use of TiO_2_ and VO_2_ as a dielectric increases the temperature-sensitivity of Ag nanoparticles and increases the wavelength tunability as a consequence of the increasing difference in dielectric functions [36]. 

**Figure 1:**
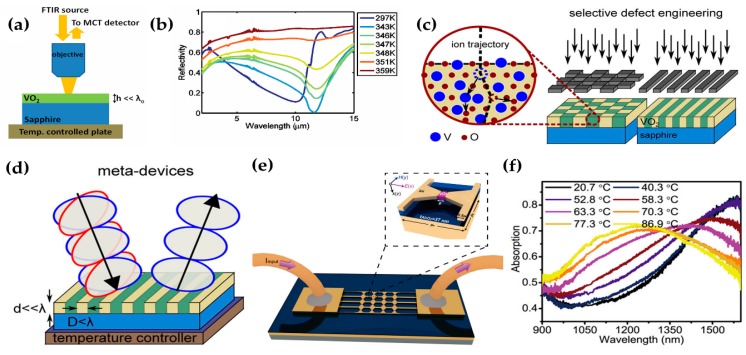
(**a**) A VO_2_ perfect absorber: The design of the material structure and experimental set up inside an infrared microscope. VO_2_ is deposited on a sapphire substrate with height = 180 nm. Temperature is controlled by a stage plate; reflectivity is measured using a mid-infrared microscope, a Fourier transform infrared (FTIR) spectrometer and mercury-cadmium-telluride detector with normal incidence. (**b**) Reflectivity spectrum at temperatures from 297 K to 360 K. Reflectivity is 0.0025 at *λ* = 11.6 μm; i.e., absorption = 99.75%. Reproduced with permission from © 2012, AIP Publishing [44]. (**c**) Schematic of engineering selective defects in VO_2_ by using ion beam irradiation with a mask. Defects in the checkerboard or striped patterns can be achieved by changing the masks. Energetic ions strike the VO_2_ layer and trigger structural defects by moving the vanadium and oxygen atoms. As irradiated VO_2_ has a lower transition temperature, the two states of VO_2_ co-exist at a lower temperature than in the undamaged VO_2_. (**d**) Stripe defected switchable polarizer: When the thickness of the VO_2_ layer is much less than the wavelength, the stripe defected VO_2_ can be used as a switchable polarizer. The polarization direction affects the absorption by the meta-device; this property provides a variable degree of optical anisotropy. Reproduced with permission from © 2016, American Chemical Society [53]. (**e**) A schematic of a bow-tie shaped unit cell that has a small amount of the VO_2_. The sizes of the structure are length = 264 nm, width = 300 nm, wire width = 100 nm, and gap width = 34 nm. A spacer dielectric layer made of Al_2_O_3_ is located between the bow-tie structure and a thick gold backplane to give a gap. Reduction in the amount of VO_2_ increases the switching speed and precision of controllability. (**f**) The temperature affects the relationship between wavelength and absorption. The tuning range is up to 360 nm (NIR). As the temperature increases, the curve shifts the peak of the absorption into the short-wavelength direction. Reproduced with permission from © 2017, American Chemical Society [52].

An ultra-thin VO_2_ layer (thickness = ~ *λ*/65 = ~ 180 nm) on sapphire functions as a perfect light absorber (up to 99.75% at *λ* = 11.6 μm) as a result of a transitional state in VO_2_ in which metallic and insulator states co-exist (Figure 1a) [44]. In this ultra-thin VO_2_, the imaginary part of the refractive index is large, so critical coupling to a cavity resonance occurs, and this process increases the metamaterial’s absorption. This MM absorber can be made easily by just depositing VO_2_ on sapphire, so the material has high potential to be used widely in optical systems.

An MM structure that uses Al as a metal, ZnS as a dielectric spacer, and VO_2_ as a material that can change its state yields a thermally-switchable tri-layer absorber that is reflective at around *λ* = 22.5 THz when VO_2_ has dielectric properties, but absorptive when VO_2_ has metallic properties; the modulation of the reflectivity levels was 58% at 22.5 THz and 57% at 34.5 THz [33]. VO_2_ conductivity changes by three to four orders of magnitude compared with the dielectric state, so when this MM is used in a metal-dielectric-metal structure (MIM), it can sustain electric and magnetic resonances simultaneously [45]. 

Au nanorods and VO_2_ film affect each other when Au nanorods are used as plasmonic arrays on VO_2_ film. Due to plasmonic enhanced light absorption and the photothermal effect, the transition temperature of VO_2_ decreases and simultaneously the plasmonic peak is changed from 685 nm to 618 nm inversely when VO_2_ is the metallic state [46]. Moreover, Au/VO_2_ absorbs light strongly, so heating Au/VO_2_ requires ~28.6% less laser power than heating bare VO_2_.

A combination of a conventional resonant MM absorber [47] with a partial VO_2_ film achieves a tunability of the absorption and resonance frequency. Unlike other research that achieved a red-shift, the Wen et al. approach realized a blue-shift, which increases the flexibility for practical applications [48]. When VO_2_ has metallic properties, it reduces the inductance of an electronic split ring resonator, so the resonance frequency increases. 

A polarization-dependent and independent structure can be achieved using a nanowire grating or nanodisc arrays, and exploiting each dielectric and metal state of VO_2_ instead of their co-existence [49]; a hybrid structure of gold-VO_2_, Au, and sapphire can be tuned according to the state of the VO_2_. When VO_2_ becomes metallic, its refractive index changes and does not show resonant behavior. Consequently, the hybrid gold-VO_2_ acts as a continuous reflective surface and the absorption is suppressed. On the other hand, when VO_2_ becomes dielectric, the structures showed high absorptivity at room temperature, with changes of up to 70% with nanowire grating and up to 64% for nanodisc arrays.

As Tungsten (W) can be used for an ultrathin absorber [50], a tunable MM absorber is achieved by combining VO_2_ and Tungsten, and it is fabricated in a square lattice nanostructure [51]. The square lattice nanostructure has a broad tunable range from 9.96% (metal state) to 99.7% (dielectric state) at 5.28 μm, but the three-layer structure does not have a tunability even though the components are the same in both structures. The tunability of the two-layer structure is achieved by excitation plasma resonance; its effect can be controlled by adjusting the period of the square lattice. A small amount of VO_2_ at the feed gap of a bow-tie field antenna (Figure 1e) reduces the size of the parts that are controlled by temperature; the heating is integrated and local, so switching can be faster and more precise at less power than in a VO_2_ thin film [52]. This meta-device has a tuning range of 360 nm for absorption and a modulation depth of ~33% with a response time of 1.27 ms. Moreover, the device was persistent during testing for >24,000 cycles.

Some methods use VO_2_ in different ways by an additional process. Selective defect-engineering has produced tunable absorbers and tunable polarizers [53]. Selective defect-engineering is achieved by ion beam irradiation (Figure 1c); after the process, the irradiated regions have a lower transition temperature than pristine VO_2_. However, the tendencies of the reflectance curves do not quite differ; this similarity means that that the selective defect-engineering does not change the original state-transition property of VO_2_. With the combination of irradiated and intrinsic VO_2_, the temperature at which the two states co-exist is lower (60 °C) than in pristine VO_2_ (68 °C). The irradiated and pristine VO_2_ have different complex refractive indices, so this combination induces anisotropy that can be tuned by adjusting the temperature. Therefore, stripes defected VO_2_ can be used as a switchable polarizer (Figure 1d). When polarized light (*λ* ~ 11 μm) parallel to the ridges is primarily absorbed at the intermediate temperature of 60 °C, only the perpendicularly-polarized light can be obtained. Therefore, this defect-engineered structure can be used in tunable absorbers or tunable polarizers that are controlled by temperature. 

A totally-encapsulated VO_2_/Au/VO_2_ composite structure has a larger modulation range than that of half-covered structure and improves the tunability of the LSPR peak [54]. In the encapsulated structure, the modulation range depends on the thickness of the top and bottom VO_2_ layers. The increase in the modulation range is caused by the thick top layer of VO_2_ due to an increase in the change in the rate of the equivalent permittivity.

### 2.2. Thermally-Responsive Material: GST

GST changes reversibly between crystalline and amorphous states when heat is applied through an optical or electrical stimulus, and it is bistable and nonvolatile [55].

**Figure 2:**
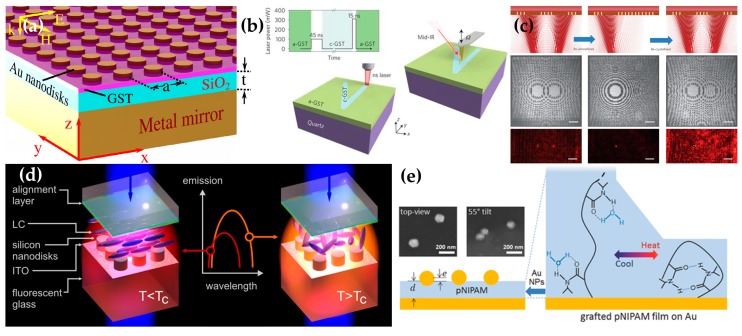
(**a**) The metal-dielectric-metal structure (MIM) structure with GST between an Au disk array and a SiO_2_ insulating layer for the tunable perfect absorber. The Au disks are 300 nm in diameter and 20 nm thick; the GST layer is also 20 nm thick. The absorption of this structure changes depending on the crystallization level. Reproduced with permission from © 2015, Chinese Laser Press [56]. (**b**) To induce change to c-GST, a 100-mW pulsed laser beam with a 45-ns pulse is used; to transform to a-GST, a 310-mW pulsed laser beam with a 15-ns pulse is used. (bottom) The schematic of the process of switching from a-GST to c-GST in V-shape with a suitable laser. (right) Scattering-type scanning near-field optical microscope (s-SNOM) image of the SPhPs excited in this structure. In this structure, the SPhPs form; they reflect from the boundary of a-, c-GST, and the process can be visualized using s-SNOM. Reproduced with permission from © 2016, Springer Nature [57]. (**c**) Dynamically optically reconfigurable zone-plate device. (top-left) Two Fresnel zone patterns of c-GST obtained using 0.39-nJ write pulses that focus a plane wave onto two foci. (top-middle) A single l.25-nJ erase pulse changes one of the Fresnel zone patterns to a-GST. (top-right), then by using 0.39-nJ pulses write pulses, the a-GST returns to the c-GST state. (middle line) Optical images of the Fresnel zone pattern on the GST film. (bottom line) At *λ* = 730 nm, red dot: transmission focal spot. The write–erase–write reconfiguration cycle is achievable by changing the GST phase (Scale bar: 10 μm). Reproduced with permission from © 2015, Springer Nature [59]. (**d**) A schematic of silicon metasurface embedded in a liquid crystal (LC). The LC is nematic at temperature *T* > 58 °C, and isotropic at *T* < 58 °C. This state change is reversible, and according to the state, the metasurface resonances wavelength shifts and the tuning range of emission changes. Reproduced with permission from © 2018, American Chemical Society [67]. (**e**) Schematic of changing the volume of the poly(*N*-isopropyl acrylamide) (pNIPAM) film. The pNIPAM film is placed between Au mirror and Au nanoparticles (NPs); the film volume changes at 30 °C and thereby alters the distance between the mirror and the NPs. (Right: magnification of pNIPAM to show the change). The transition temperature is ≈30 °C. Insets: Scanning electron microscope (SEM) images of this structure from different angles. Reproduced with permission from © 2016, John Wiley and Sons [68].

A 20-nm-thick GST layer in a metal/dielectric/metal (MIM) structure (Figure 2a) achieves a 650-nm tunable range for the absorption according to a crystallization level of GST (0% for amorphous phase and 100% for crystalline phase) [56]. This tunability occurs because the refractive index is higher in crystalline GST (c-GST) than in amorphous GST (a-GST), and this difference affects both the resonant wavelength of the upper metal part (Au disks) and the impedance of the structure. Therefore, a higher crystallization level of GST causes an increase in the wavelength at which perfect absorption occurs. 

An ultrathin GST film on quartz enables dynamic control of surface phonon-polaritons (SPhPs) with single laser pulses, and production of ultra-confined SPhPs (polariton wavevector is 70 times larger than the wavevector calculated by 2*π*/*λ*) in quartz [57]. The c-GST region is brighter than the a-GST region at a frequency of 943 cm^−1^ (no SPhPs) and the a-GST region has strong SPhP fields at a frequency of 1135 cm^−1^ (with SPhP excitation). A nanosecond laser beam can change the GST phase by rapidly depleting electrons from resonantly-bonded c-GST [58]; this phenomenon can be exploited to write and erase shapes selectively in certain regions by changing between a-GST (written) and c-GST (erased) (Figure 2b). However, the resonance strength weakens as the number of switches increases. 

Several dynamically reconfigurable optical devices have been obtained using GST film; these include binary and greyscale devices, writing planar wavelength multiplexing focusing devices, a dynamically- and optically-reconfigurable zone-plate device (Figure 2c) and writing a dielectric MM [59]. Although the conventionally nanosecond and microsecond laser pulses can induce phase change materials to switch their states between amorphous and crystalline phases in optical data storage technology, femtosecond laser pulses are recently verified that it can induce multi-level switching [60,61], so the authors use femtosecond laser pulses to induce a GST phase transition with a change in the refractive index in an extremely small volume of 0.02 μm^3^. The complex refractive index changes in accordance with the energy and the number of stimulating optical pulses, so they find writing and erasing conditions (‘Write’ channel) and observe these patterns by using a charge-coupled device camera at *λ* = 633 nm (‘Read’ channel). 

An ultra-broadband polarization-independent MM perfect absorber that consists of GST array, silica, GST cavity, and Au substrate has been analyzed numerically; the temperature of GST parts could be increased to 433 K in 0.37 ns or to 481 K in 0.56 ns with a light intensity of 1.11 × 10^8^ W/m^2^ [62]. This rapid change is due to strong plasmon resonances on the array and to cavity resonance.

The emissivity of polar materials has been enhanced by using gratings [63], metasurfaces [64], and tunable materials such as graphene [65] or VO_2_ [52], but these devices have limitations such as a low emissivity, complex fabrication process, high energy requirement, and insufficient chemical stability. GST film on a silicon carbide SiC polar crystal overcomes these drawbacks and achieves a dynamic control of emissivity, but needs only a deposition process; this emitter has an angular insensitivity, zero-static power, and a ratio of emissivity to extinction >10 dB over the Reststrahlen band of SiC (11.3 μm to 12.3 μm) [66]. The GST states affect the characteristics of the distributions of electric fields and resistive loss.

### 2.3. Others

Instead of VO_2_ and GST, other materials can be used as elements of tunable metamaterials. Here we introduce three additional materials and how they change with temperature. 

Liquid crystals (LCs) have also been used as a thermally-tuned material. A Mie-resonant dielectric metasurface is embedded in an LC that can switch its state depending on the cell temperature (Figure 2d) [67]. When heated to higher than a critical temperature *T*_C_ = 58 °C, the LC state changes reversibly from nematic to isotropic. The difference of the local photonic density for each state leads to shifting resonances position, so this metasurface realizes a reshaping of the emission spectrum as well as the dramatic change of the transmittance spectrum owing to the Purcell effect.

Poly(*N*-isopropyl acrylamide) (pNIPAM) is a polymer that shows a strong volume shrinkage (up to 90%) above the critical hydration temperature (≈30 °C) and it causes a color change from green at 25 °C to orange at 35 °C, which is different from previous structure colorings [69,70]; the transition is completely reversible. This transition has been exploited to make a fast and reversible plasmon-tuning system [68]. As shown in Figure 2e, the pNIPAM is placed between Au nanoparticles (Au NPs) and an Au mirror (Au film) and modulates the plasmonic resonant modes by controlling the distance between scattering NPs and film. 

Distilled water (DW) has been used as a thermally changeable component in MM absorbers [71]. DW has the advantages of frequency-dispersive characteristics and a large imaginary part in its permittivity, but DW causes the mismatch of impedance over broad frequency region. To overcome that challenge, the DW was held in a dielectric reservoir and a copper backplane was used. The thickness and height of DW and the arranged dimension of water-substrate determine many factors that affect absorption, including impedance, absorption bandwidth, and a peak of the high frequencies. A metallic fishbone structure on the side of a DW substrate improves broadband absorption and achieves an ultra-wide absorption bandwidth with thermal tunability by exploiting the temperature-dependence in the permittivity of DW. 

## 3. Optically Driven Tunable Metamaterials

Optical elements change depending on what optical source is used and its strength. Optically-tunable MMs generally have the advantages of fast modulation speed and a tunable range in the visible spectrum, and compatibility with integrated circuits. However, they have the disadvantage of a narrow tunable range, and it cannot be adjusted for each cell, but only for the entire structure. The optically tunable MM will contribute to high-capacity communications, real-time holograms, and adaptive optics via the fast and reliable response (MMs that are heated by light to change their properties are classified as thermally-tunable in this review).

### 3.1. Optically-Responsive Material: Indium Tin Oxide

ITO is a material of an optically-tunable MM; its permittivity can be changed by several factors, including plasma frequency, charge carrier collision rate, the carrier concentration, and the effective mass of the electron [72]. Therefore, a pumping laser can affect these factors and activate an optically-tunable MM that contains ITO. 

An antenna-ITO hybrid system that combines a gold nanoantenna with indium tin oxide (ITO) can achieve a picosecond response [73]. In this system, a pump laser generates plasmon-induced hot electrons, which are injected from the Au nanoantenna into the conductive ITO; this change amplifies its large free-carrier nonlinearity. 

**Figure 3:**
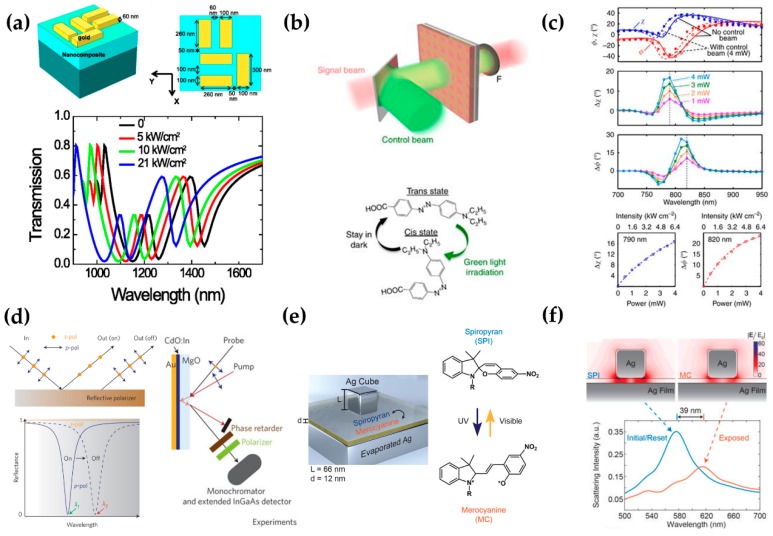
(**a**) The schematic structure of the meta-molecule with a top-view. The meta-molecule consists of Au nanocuboids and a nanocomposite that provides a large third-order nonlinear susceptibility. The nanocomposite is composed of polycrystalline indium-tin oxide doped with Au nanoparticles. The bottom graph is the calculated transmission spectra of this MM according to pump intensity. With a weak pump laser (21 kW/cm^2^), the tuning range is 120 nm. Reproduced with permission from © 2014 AIP Publishing LLC [74]. (**b**) A schematic of how to operate the structure. The control beam is green light (532 nm) which interacts with a photoisomerizable azo ethyl red to cause a switch in the polarization effects of the metasurface. (Top) The single beam combined with control beam by a dichroic mirror passes through the nanostructure and a long pass filter that allows only a single beam to pass. (Bottom) Structures of trans and cis state of the ethyl red molecule. Green irradiation causes a change from the trans to the cis state; darkness allows a return to the trans state. Reproduced with permission from © 2017, Springer Nature [79]. (**c**) A graph of polarization parameters. Polarization parameters (ϕ, χ) measured from the transmitted beam; dots: measured data; lines: simulation results. When the changing ethyl-red molecule changes from trans to cis, both polarization parameters shift toward blue. Depending on the control light power, the measured parameters show nonlinear changes (Δϕ and Δχ), and each parameter is compared with the results without the control beam. Irradiation with 4 mW green light (*λ* = 820 nm) induced a nonlinear change Δϕ ~23.2° in the transmitted polarization azimuth. (**d**) A schematic of a switchable reflective polarizer. It consists of an indium-doped cadmium oxide (CdO:In) that has a high carrier mobility, an Au capping layer and a magnesium oxide (MgO) substrate. An unpolarized incident beam is reflected by the polarizer and the output beam can have different polarization states depending on whether the polarizer is on or off. Bottom graph: concept of how the polarizer works. When the polarizer is turned on or off, the reflection spectrum of the p-polarization (p-pol) beam changes. When it is switched on, the polarizer acts as a perfect absorber for the p-pol at wavelength λ_1_; when the polarizer is switched off, it acts as a mirror for p-pol at λ_1_. Right: set-up of the pump-probe measurement. The reflectance of p-pol is changed due to the photoexcitation of the CdO film, and this change is dependent on the energy density of the pump beam. Due to the changing reflectance, the degree of p-pol state of the reflected light varies with wavelength. Reproduced with permission from © 2017, Springer Nature [80]. (**e**) A schematic of the spiropyran (SPI)/PMMA layer is integrated into nanopatch antennas and changing the chemical structure of SPI. The SPI/PMMA layer is integrated into nanopatch antennas comprised of silver nanocubes and silver film (left). The chemical structure of an isomer of SPI changes reversibly between Spiropyran and Merocyanine (right). Depending on the types of light, the C-O ring opens or closes and this affects the optical properties of the isomer. Reproduced with permission from © 2017 American Chemical Society and https://pubs.acs.org/doi/pdf/10.1021/acs.nanolett.7b04109. Note that further permissions related to the material excerpted [81] should be directed to the ACS [81]. (**f**) The simulated electric field increase of the fundamental plasmonic mode (SPI: 565 nm and MC: 610 nm) and experimental data about the scattering intensity. Initially, the reset resonance wavelength is 577 nm and after ultra-violet (UV) exposure, it red-shifts by 39 nm to 616 nm and the full width at half maximum decreases to 0.75 times the initial value.

Tunable double plasmon-induced transparency has been achieved by placing gold nanocuboids on a nonlinear nanocomposite film made of polycrystalline ITO doped with Au nanoparticles (nano-Au:polycrystalline-ITO) (Figure 3a) [74]. The double transparencies are obtained by the destructive interference coupling between two superradiant modes and one subradiant mode. A large third-order nonlinear susceptibility is also obtained as a result of three nonlinearity enhancement factors: the local-field effect that originates from the nonuniform electric field distribution of the pump laser [75], the quantum confinement effect [76], and a hot-electron injection from Au nanocuboids to the nanocomposite [77]. This MM has a 120-nm tuning range under weak laser pumping (21 kW/cm^2^) that is five orders of magnitude lower than the conventional case because of the highly nonlinear susceptibility of the nanocomposite. The response time is 34.9 ps. 

An ultrafast all-optical tunable chirality with ultralow-power is achieved by designing L-shaped gold nano-antennas twisted 90° to each other with a large nonlinear nano-Au:polycrystalline-ITO layer; the chirality could operate with circularly-polarized light [78]. Circular dichroism is a result of different plasmonic resonant modes excited by the reverse rotation of the electric field vectors in two circularly-polarized incident waves. The circular dichroism attains maximum value (30%) at *λ* = 1370 nm, but *λ* at which circular dichroism is greatest, decreases as much as 45 nm in 35 ps when this tunable MM is excited by a weak 40 kW/cm^2^ laser pump. The pump laser causes a decrease in the refractive index of the nonlinear layer, so the resonance wavelength of the plasmonic modes shifts toward blue.

### 3.2. Optically-Responsive Composites 

Conventional polarizers, such as a wire-grid polarizer, birefringent crystal, and apparatus using electro-optical or acousto-optical effect, are either static or possessing the only nanosecond response times, but a gateway plasmonic material composed of indium-doped cadmium oxide (CdO:In) that has a high carrier mobility, an Au capping layer and magnesium oxide (MgO) substrate has yielded a reflective polarizer that achieves fast tuning (800 fs) with a range from near- to mid-infrared frequencies [80]. Optical pumping at with 339 μJ·cm^−2^ shifts the perfect absorption resonance strongly toward the red, so p-polarized reflectance changes from 1.0 to 86.3% at 2.08 μm and from 73.0 to 10.5% at 2.23 μm (Figure 3d). The intraband optical pumping can alter the electron effective mass in the plasmonic material by creating a hot-electron distribution; the time required for the effective mass change is influenced by the heat exchange rate between the electrons and the lattice. Due to the shifting p-polarized reflectance, the degree of the reflection of p-polarized light depends on the wavelength.

Metallic split-ring resonators with incorporated semiconductors can achieve a tunability by changing the pump power [82]. The split gap at the center of the unit cell is considered as a capacitor. Since photoexcitation changes the effective size of the capacitor causing conductivity changing, this enables tunability. As the photoexcitation influence increases, the resonance frequency that depends on the capacitance can decrease monotonically, so this MM can be frequency-tunable in the terahertz range with a tuning range of 20%.

Rearranging the structure of Au nanocuboids, changing the nanocomposite to polycrystalline barium-titanate-doped gold nanoparticles (nano-Au:polycrystalline-BaTiO_3_), and covering the nanocomposite with a multilayer tungsten disulfide (WS_2_) micro sheets decreased the threshold pump intensity to 20 MW/cm^2^, which is lower than those of previous reports [83,84,85,86,87,88,89]; the response time of this structure was 85 ps due to fast carrier relaxation dynamics in the nanocomposite [90]. The nanocomposite and WS_2_ act as nonlinear optical materials and their optical nonlinearity are amplified by the quantum-confinement effect, the field-reinforcement effect, and the local-field enhancement effect caused by the non-uniform electric-field distribution of a pump light. Because of this increased optical nonlinearity, the MM provides a large third-order nonlinear susceptibility and transmission spectrum that varies with the intensity of the pump light.

### 3.3. Optically-Responsive Polymers

A layer of nonlinear azobenzene polymer (poly((methyl methacrylate)-co-(disperse red 13 acrylate))) on periodic arrays of an asymmetrical square lattice of meta-molecules etched in a gold film has achieved a shift of Fano resonance wavelength by a weak pump light [76]. The azobenzene polymer’s refractive index changes greatly as a result of extremely enhanced photoisomerization associated with resonant excitation, so this MM provides a large optical nonlinearity. The tuning range of the Fano resonance wavelength is maintained with a weak pump light (0.06 kW/cm^2^), which is seven orders of magnitude lower than that of conventional optical materials (GW/cm^2^). 

A metasurface composed of a periodic array of gold L-shaped slits and a thin layer of photoisomerizable azo ethyl red as a switching layer provided nonlinear changes in the polarization azimuth of a transmitted beam at optical frequencies [79]. Under a green incident light, the ethyl red molecule changes from a trans state to a cis state, then under darkness, it returns to the trans state (Figure 3b). Due to this change property, the molecular polarizability and the refractive index of the polymer layer change according to the Lorentz-Lorenz-condition [91]. The resonant plasmonic modes are coupled, and the ethyl red layer can switch between two isometric states, so 4 mW of green light induces >20° nonlinear change in the transmitted polarization azimuth.

A composite made by photoisomerization of spiropyran (SPI) and poly (methyl methacrylate) (PMMA) as the spacer layer can tune plasmon resonances and overcome the severely limited tuning range of conventional tunable MMs [81]. UV irradiation causes the SPI to isomerize to merocyanine (MC); the change can be reversed by visible light or heat exposure (Figure 3e). The MC isomers are coupled to the plasmonic resonance, so the scattering spectra of this MM red-shift during UV exposure (Figure 3f). This device shifted the plasmon resonance by up to 71 nm (i.e., a figure of merit of 1.43). 

## 4. Structurally Deformable Metamaterials

Many types of MMs or metasurfaces have unique properties that are a consequence of the arrangement of the structures, and the shape of each element. Thus, the properties of these MMs can be tuned by applying an external excitation that deforms the shape or arrangement of a structure; if the deformation is continuous, the change in properties change may also be continuous. Most methods of imposing such a deformation fabricate nanostructures on the stretchable substrate then stretch it, or fabricate a MEMS MM device and apply a voltage. Magnetoelastic, strain field, piezoelectric properties can also be used to obtain tunability by mechanical deformation.

### 4.1. Stretchable Substrates

**Figure 4:**
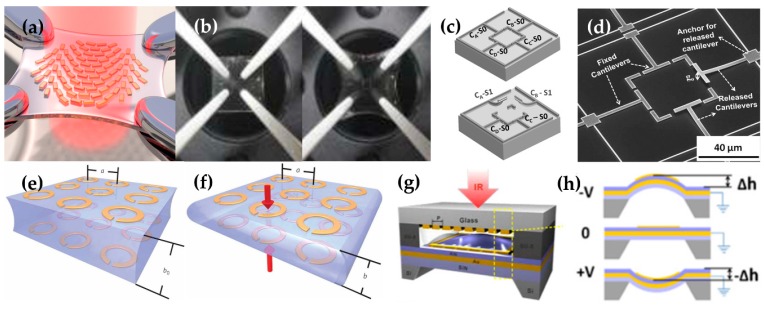
(**a**) The schematic of tunable metalens fabricated on an elastic substrate. The four corners of the elastic substrate are held by the arms, and when the force is applied in four directions, the elastic substrate is stretched and the spacing of the gold patterns changes. (**b**) Photograph of the fabricated metalens before and after stretching. Reproduced with permission from © 2016, American Chemical Society [92]. (**c**) A schematic of un-activated and activated MEMs tunable MM. The voltage applied to each arm in the four directions can release the arm from the surface. (**d**) A SEM image of the pattern. Reproduced with permission from © 2014, AIP Publishing LLC [93]. The cantilever on the right and bottom side have been released. A schematic of MM (**e**) before and (**f**) after transformation. Reproduced with permission from © 2017, Springer Nature [94]. When the magnetic fields are applied to the patterns on the top and bottom side, a force that pulls each side is generated. As a result, the elastic interlayer substrate shrinks, so the properties of the MMs change. (**g**) A schematic of total material, (**h**) Schematic of the tuning mechanism; depending on the applied voltage, the structure is bent up or down by piezoelectricity, so the volume of the voids changes and the property of the entire structure is tuned. Reproduced with permission from © 2016, Optical Society of America [95].

A flat optically-tunable meta-lens can be achieved by the patterning of a gold metasurface on stretchable polydimethylsiloxane (PDMS) substrate (Figure 4a,b) [92]. It operates in the visible frequency regime, and stretching the lens enables continuous tuning of the optical wavefront. Stretching widens or narrows the gaps between gold nano-size patterns; as a consequence, the spatially-varying phase discontinuity term in the generalized Snell’s law is modulated. The phenomenon enables control of the properties of the lens, including the anomalous refraction angle. This study overcame the limitation of flat lenses fabricated from metasurfaces that have a single fixed characteristic. The study also advanced the field of formal stretching-tunable MMs, which has been focused on tuning individual optical resonators and their properties, not on tuning entire surfaces and optical wavefronts. This addition of tunability to MMs has advanced them toward practical applications. 

Early research on tunable MMs on stretchable substrate involved measurement of Raman scattering while controlling the space between two gold nanoparticle dimers on an elastomer [96]. The sophisticated control of nano-gaps is challenging, but these tunable MMs allow for continuous control of the gap size, understanding of plasmonic coupling at the nanoscale has been advanced. Raman scattering between gold nanoparticles with a radius smaller than the wavelength can be clearly observed from the dark-field scattering spectra at different stretching distances; these spectra are anisotropic with the polarization direction. This study considers several nanoparticles and their properties and shows that spacing and consequent tunable properties can be controlled simply by using a stretchable substrate. Early research looked at only several nanoparticles and their tunable properties, but some studies show the feasibility of tuning a metasurface, which is a repeating set of nanopatterns.

The working frequencies of structures can be controlled about 400 nm by making several types of split ring resonator (SRR) structures on a flexible substrate, then stretching it to adjust the gaps between the structures [97]. This stretching can be used to tune the optical characteristic of the entire surface over a broad range. The SRR structures can be modeled as an *LC* resonator with resonant frequency ω_0_ ~ (*LC*)^−1/2^, where capacitance *C* depends on the gap size and inductance *L* is propositional to the total effective length of each SRR. Therefore, it is possible to control the ω_0_ of the *LC* resonator by controlling the gap. The work demonstrated SRR-bar, asymmetric coupled SRR, square SRR, and double SRR arrays. The metallic elements on the stretchable substrate could withstand a tensile strain as high as 50% with distortion. 

An elastomer can be used to form a reversibly-wrinklable metasurface [98]. This research suggests that the mechanical deformation of the elastomer with a metasurface is not limited to two dimensions but can have tunability from a wave-like winkled structure to a planar structure. This paper confirmed the feasibility of gaining sufficient robustness and variation in characteristics of tunable MM. Their structure was fabricated using a method based on ‘wrinkling instabilities’ which uses pre-strained layers of a rigid film composite, and a soft backing layer that relaxes spontaneously. Likewise, adding a stretchable substrate to existing MMs make it possible to tuning the working frequency of MMs and even can adding special properties into existing MMs. 

Mechanical deformation can also be used in acoustic MMs. An acoustic MM in which the structure rotates in response to applied strain achieved a rotational transformation rather than a linear change in the unit structures [99]. The resonating mass is supported by easy-to-buckle elastic beams, rather than by an elastomer. A uniaxial strain causes the array of beams to buckle, so their stiffness changes, as do the natural frequencies of the resonating units. 

Materials research in this area is also developing. A dielectric resonator on conductive rubber as a stretchable substrate achieved near-unity absorption around the magnetic Mie resonance; the working frequencies of this device can be tuned [100]. The decreasing longitudinal coupling effect between adjacent dielectric resonators cause the absorption peak to blue-shift by 410 MHz. This work considered only two dielectric bricks and their resonating features, but it demonstrates the feasibility of using a new type of stretchable material and adapting dielectric materials into tunable MMs. 

Tunable metasurfaces can be fabricated in all-dielectric materials rather than conductors [101]. This work is a large advance over [100], which used only two dielectric bricks. Fabricating a dielectric nanostructure onto PDMS was difficult, but they produced a tunable all-dielectric metasurface with a smaller loss than metasurfaces that use conductive materials. PDMS, as the soft substrate in their structure, allowed tuning of the period of TiO_2_ resonators which are a hard material. Resonance shifted due to the tuning of the period. To interpret the resonance frequency shift, which their structure can exert, they analyze the near-field interactions with a Lagrangian model. 

Another tunable meta-lens [102] has a tunable focal length from 600 μm to 1400 μm, which means that the ratio between the minimum and maximum focal lengths is more than 2. The lens is constructed of encapsulated amorphous silicon nanoposts (meta-atoms) that have a high refractive index *n* in a dielectric in a subwavelength periodic lattice in PDMS which has low *n*. This high contrast in *n* causes weak optical coupling, and this weak coupling causes a high localization of energy density inside the nano-posts. This weak optical coupling between the nano-posts is a key characteristic in the design of their tunable meta-lens. 

Because ordinary meta-holograms have one image at first demonstration, attempts to implement multiple images in one metasurface have been made [103]. However, the meta-holograms still have a fixed number of images. A tunable meta-hologram [104] solves the problem that an ordinary meta-hologram can only show a limited number of images. They fabricate anisotropic gold nanorods on PDMS. Position-dependent phase discontinuity which stems from the orientation angle of the nanorod-created hologram images. Structural changes due to isotropic stretching changed the hologram image. Isotropic stretching of the metasurface altered the position-dependent phase discontinuity and reconfigured the optical wavefront. They employ Fresnel transformation to rigorously understand how a wavefront changes.

Rearrangement of structures by deformation can yield the full-color range on one metasurface [105]. The pattern consists of a periodic array of rectangular aluminum nanostructures. Aluminum has a high bulk plasma frequency, so its plasmonic resonance is tunable from the ultraviolet to infrared. The far-field interference among unit cells in the lattice suppresses the scattered light outside the target tuning range and thereby provides vivid colors. Stretching the metasurface changes the period of structures and their resonance features, so the metasurface shows different colors when stretched in different directions.

### 4.2. Micro-Electro-Mechanical System

A micro-electro-mechanical system (MEMS) is a small device that is regulated by electrically-induced physical deformation or movement. 

A tunable split-ring resonator structure (Figure 4c,d) [93] can be directly physically deformed by an induced voltage so that the property can be controlled at THz frequency range. Two of four cantilevers can be actuated in out-of-phase directions, while the other two cantilevers are fixed. Depending on which cantilever is released, resonance features that can be explained by the *LC* model are changed. Each unit cell has multiple movable cantilevers, so various combinations of changing properties are possible. In tunable MMs that use MEMS, the unit cell size is usually somewhat large to allow verification of change or movement, so the device has a rather long operating wavelength. 

A switchable structure is a relatively simple tunable metasurface that uses MEMS. Two modes of MM can be implemented with a MEMS switch structure [106]. The unit cell can have an on and off mode, so that the unit cell can assume a resonance state or a nonresonance state, thereby obtaining tunability. This study demonstrated that the MEMS structure can be used as an MM and is tunable. The unit cell is large, so the working frequency is in the gigahertz frequency range. 

Arrays of switches with SRR structures [107] allow the SRR structure to be in the off-state or the on-state. The research shows that MEMS reconfigurable MM can be realized on a surface level, and is not restricted to the unit cell level. The device uses low-loss quartz. The structure consists of a square SRR and a cantilever with a disk inside. When the voltage is not applied, the cantilever with the disk is suspended; when a voltage is applied, the disk contacts the surface with a small quartz gap relative to the size of the disk. As the gap decreases, the *LC* resonance changes, so the terahertz transmission can be tuned. In the ‘off’ state, the device has a resonance frequency at 696 GHz, and in the ‘on’ state, it has resonance frequency at 461 GHz in the electric field parallel to the MEMS-SRR cantilever. The electric field perpendicular to the MEMS-SRR cantilever showed no resonance, so this device achieves polarization selectivity. 

A MEMS comb-drive structure yielded an MM structure with a continuously-controlled gap [108]. The gap of the MEMS comb-driver structure is precisely controlled by an applied voltage. When the gap of the MEMS comb-driver structure is precisely adjusted, the gap between the MMs connected to the comb-driver is also precisely controlled. The gap size can be controlled from 1 μm to 10 μm continuously. Therefore, this MM filter can have a large tuning range. For incident light, the size of the gap affects the distributions of the magnetic and electric fields, so the optical response changes. This structure can achieve continuous tuning of the properties by the continuous movement of the structure; in this sense, this structure differs from the switch structure.

MMs with structural anisotropy exhibit different responses that depend on the polarization direction of incident light. A MEMS MM that is tunable and dependent on polarization direction is possible [109]; the structure consists of a square unit cell with a pair of cantilever-shaped arms inside. These arms are lifted according to an applied voltage, and the properties are changed accordingly. Here, the reaction of the structure differs according to the polarization direction of the incident light, so the device can be tuned without disturbing this property. 

### 4.3. Magnetoelastic, Strain Field, Piezoelectricity Modulation

Mechanical deformation can be used to tune MMs. In one such device, the distance between multilayer structures is reduced by electromagnetically-induced forces (Figure 4e,f) [94]. The magnetoelastic MMs consist of two layers of SRR arrays or capacitively-loaded rings. The induced magnetic field H_0_ in the axial direction imparts resonant magnetic behavior to the MMs. The resonators affect each other by mutual inductance and attractive Ampere force when magnetic fields are introduced. By those interactions, the MMs’ properties are modulated by the thickness of the intermediate layers. This study differs from the studies mentioned earlier in that the thickness of the intermediate elastomer layer is reduced by the magnetically induced force, rather than by mechanical stretching, and that the electric force is induced by an electric potential. Another difference is that the inducing force and modulating properties are realized by the same-shaped two-layer MM. 

The strain field generated by the induced voltage can be controlled; this ability can be exploited to make a tunable meta-lens that controls astigmatism, shift, and focal length [110]. This tunable meta-lens is a combination of metasurfaces used in general meta-lenses, and dielectric elastomer actuators (DEAs), which are referred to as ‘artificial muscles’ in soft robotics. This meta-lens design uses a hyperboloid phase profile, which is used in many other meta-lenses. However, the meta-lens divides the lens into four parts and an individual strain field is applied by DEAs to each part, so the arrangement allows for the control of the size of the lens, along with many other functions such as defocusing, controlling astigmatism, and shifting the focal point. 

Piezoelectricity can also be used to induce mechanical deformation that can be used to tune the properties of the MM. Piezoelectricity can be used to change the size of the optical cavity in a tunable MM (Figure 4g,h) [95]. The MM consists of an optomechanical cavity with an array of nanostrip antennas. Piezoelectric actuation induces a voltage, which modulates the size of the optomechanical cavity; therefore, the resonance wavelength of the perfect absorber is tuned.

## 5. Electrically-Tunable Metamaterials

Electromagnetic tuning induces changes in an MM’s electromagnetic properties. Two methods have been mainly explored. The first is to control the number of electrical carriers in the target substrate; the second is to tune the overall characteristic by adjusting the band gap of graphene. Advanced tunability can also be obtained by designing isotropic 2D structures and using liquid crystals.

The voltage applied to a particular material can increase the number of electrical carriers in it, and this increased carrier density affects its optical properties. These effects have been exploited to tune MMs. A representative example of such a material is indium tin oxide (ITO).

### 5.1. MIM Structure

The refractive index of a MIM structure can be tuned by adjusting the number of carriers in the ITO layer in it [111]. Their architecture is a metal-transparent conducting oxide (TCO)-semiconductor heterostructure, in which the TCO is the active material. When the voltage is applied, the electrical carrier concentration at the interface between dielectric and conducting oxide interface increases by more than 10 times. As a result, the refractive index in the visible spectrum changes significantly. This study demonstrates that a structure that uses ITO is integrated with an MM can be tuned electrically. 

**Figure 5:**
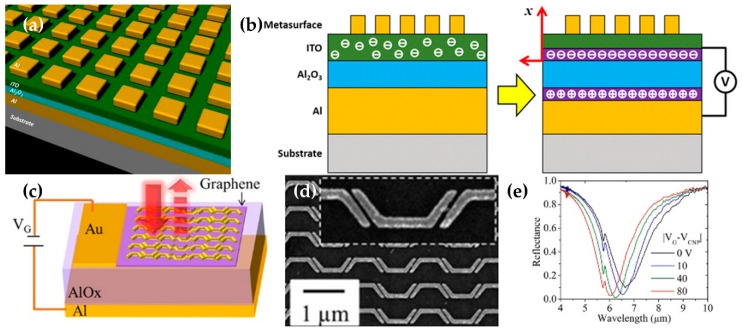
(**a**) The schematic of MIM with tunable ITO MM, with a basic MIM structure of aluminum square patterns in the top layer and an ITO layer between the top metal patterns and the insulator (Al_2_O_3_). (**b**) A cartoon of the mechanism of tuning MM by an ITO-induced structure. Reproduced with permission from © The Author(s) 2017 [113]. The voltage between ITO and metal layer increases the carrier accumulation; as a result, the reflectance change reaches 5.16 at wavelength = 2.56 μm. (**c**) A schematic of a graphene-added tunable MIM MM structure and (**d**) a SEM image. A MIM structure with patterning on the top layer makes the reflectance close to zero at a certain wavelength band. By putting a graphene layer in the middle of this structure and applying a voltage to that layer, the target wavelength band shifted. (**e**) Simulation of the reflectance change when the voltage was changed from 0 to 80 V, and the consequent change in the target frequencies. Reproduced with permission from © 2014, American Chemical Society [115].

A tunable absorber that works in the mid-infrared wavelength band can be assembled from several materials [112,113,114]. All of these papers tuned the absorption of the MM by adjusting the carrier density in the ITO layer. One of these electrically-tunable MMs consists of metal-insulator-metal with an ITO layer between an insulator and a metal pattern (Figure 5a,b). To maximize the absorption property, a metal square patch array was fabricated on the top of the device. They derived the carrier concentration of the ITO layer according to the applied voltage using a conventional metal-oxide-semiconductor model and complex permittivity of the ITO layer according to the carrier concentration using the Drude model. As the applied electric field increased the carrier concentration, ITO became metallic, and its complex permittivity changed. As a result, the minimum point of the reflectance spectra blue-shifted by 50 nm. Each paper used a different material between the metal layer and the ITO layer; this difference demonstrates that research into materials is ongoing in this field.

A MIM grating structure with ITO can be used for beam steering [116]. The tunability of their structure is a result of field-effect modulation of the refractive indices of the conducting layer and the metasurface antenna elements. The authors added independent addressability to the grating antenna metasurface to enable the electrical switching of first-order diffraction beams. The wave phase and amplitude of reflected light could be dynamically controlled by adjusting the periodic voltage applied to the grating. 

The carrier concentration and refractive index of the ITO layer of a MIM structure have been controlled to steer reflected beams [117]. The metasurface of the antennas was designed to be a square patch. By adjusting the voltage applied to each rectangular unit cell, a beam could be steered in two dimensions. 

A tunable metasurface can be produced by using an all-dielectric patterning rather than a metal in the top layer of a MIM structure; i.e., a dielectric-TCO-insulator-metal structure [118]. Here, the tuning principle also entails adjusting the carrier concentration of the ITO layer; the difference is that the current paper uses an all-dielectric metasurface and their reflection characteristic when two closely-spaced electric and magnetic resonances occur. By adopting multigate biasing, the authors achieved a relatively high reflection amplitude of 0.4 over 180° in the NIR spectrum. This study suggests that the selection of materials used in tunable ITO MMs can be expanded.

### 5.2. Graphene

Graphene is a hexagonal array of carbon with single-atom thickness and is a zero-bandgap semiconductor. It has been used to create MMs and to add tunability to existing MMs. The electrical and optical properties of a material can be tuned using electrical gating, which is induced by applying a voltage to graphene [119,120]. 

The reflectance and transmittance of graphene samples on a SiO_2_/Si substrate change significantly as a function of the gate voltage as a result of band structure changes in response to electrical gating which analyzed using the Drude model. Field-effect-modulated electrical conductivity of monolayers and bilayers of graphene yield interband optical transitions [120]. The interband optical transition causes a distinct change in optical response. This property of graphene has been used to obtain the tunability of existing MMs. 

The optical plasmonic resonance of gold nanorods in the NIR can be changed by surrounding it with graphene and applying a voltage [121]. Gold nanorods are of fixed size, so they have a fixed resonance frequency, and the intensity of their plasmon resonance is very weak. When graphene contacts the gold nanorods structure, the resonance frequency, quality factor, and resonance scattering intensity of gold nanorod plasmons can be controlled simultaneously. These changes are consequences of changes in the real and imaginary parts of graphene’s dielectric constant. 

The operating range of MM can be tuned by adding a graphene layer to the metasurface; this layer acts as a plasmonic antenna (Figure 5c,d) [122,123]. The carrier density and the Fermi level can be changed by applying a gating voltage to the graphene, and permittivity is dependent on carrier concentration. By exploiting this property, the authors turned the plasmonic antennas by controlling carrier concentration. Applying a voltage has no effect on the spacing between the structures, but gating in the graphene layer affects the electrical properties and consequent optical properties. Therefore, the tunability of the metasurface can be achieved. In most cases, graphene has been introduced into existing MMs to add tunability. 

Graphene itself can be used as a material for a tunable metasurface [115]. Arrays of closely packed graphene nanodisks with different gap and diameter show the absorption of graphene structure is tunable. A graphene layer alone interacts very weakly with visible and near-infrared light (2.3% absorbance). Nano-patterning by graphene induces localized surface plasmonic resonance, which increases the absorbance and obtaining tunability by electrical gating. 

The working frequency of a perfect absorber can be adjusted by fabricating a metasurface that includes a graphene layer between the pattern and insulator layer of a MIM absorber [115] and can be modeled as an asymmetric Fabry–Perot resonator. The perfect absorption property of the structure is also implemented when the graphene layer is not present, but adding a graphene layer and adjusting the carrier density by applying voltage yields a wide tuning range (5–7 μm) in the near-infrared region. 

The working frequency of the perfect absorber using graphene can also be used to create a dual-band MM perfect absorber by using patterns of two different sizes and shapes [124]. This dual-band MM absorber has two absorption bands that can be tuned independently. It also operates at a wide range of incidence angle.

### 5.3. Structure MMs

Some MMs exploit structural specificities while controlling the carrier concentration of the material or gating the graphene structure. Two-dimensional multi-material nanowire designs are representative examples.

**Figure 6:**
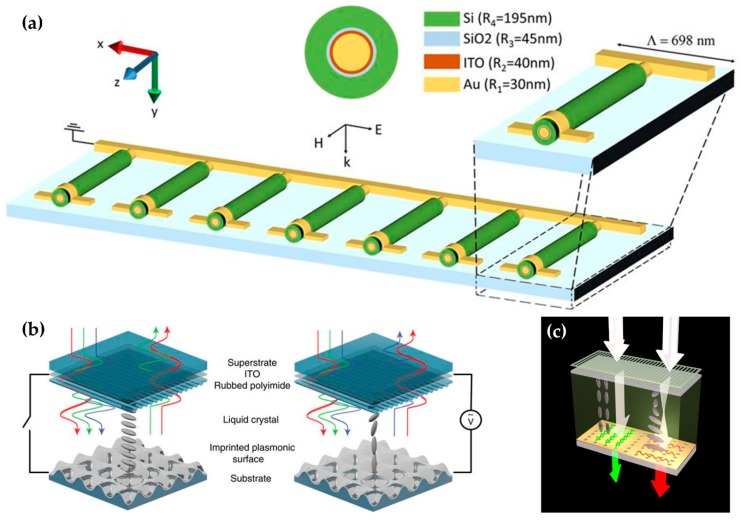
(**a**) The schematics of the cross-section and total structure of 2D tunable MMs. Reproduced with permission from © 2017, Springer Nature [125]. The array of a 2D layered line structure can transmit light. The line is composed of a metal-oxide-semiconductor integrated with ITO, which is conventionally used in tunable MMs. (**b**,**c**) tunable color-generation and color-filtering MM using the *LC* model. The alignment structure of the *LC* is changed by the applied voltage and changes the color produced by the MMs. MM in (**b**) tuning color of reflected light; (**c**) transmitted light. Reproduced with permission from © 2017, Springer Nature [127], Reproduced with permission from © 2017 American Chemical Society [128].

Tunability can be obtained by adding ITO to a metal-oxide-semiconductor and metal-insulator-metal ‘nanowire’ structure [125,126] (Figure 6a). Fabrication of the MM in this 2D form enables tuning of controlled transmitted light. The MM consisted of a set of vertical MIM nanowires; this architecture yielded the tunability and multifunctionality of MMs that had a graded refractive index. The authors also used materials other than ITO to study the tuning of the effective permeability and the effective impedance while maintaining the nanowire structure. 

### 5.4. Liquid Crystals

The properties of LCs are changed by an applied voltage, and their optical properties change accordingly. LCs can produce a spectral shift of plasmon resonance of Au nanoparticles [129]. When an Au nanoparticle was embedded in a liquid crystal, the application of changed the direction of the LC, so the anisotropic refractive index of the embedding media changed. This change results in a shift in the spectrum of the particle plasmon resonance. 

An MM that can tune color generation or filter colors has been developed by exploiting this changing property (Figure 6b,c) [127]. Both studies are similar, but Reference [127] tuned the reflected beam color by using a reflective plasmonic surface with a nanowall unit cell, whereas Reference [128] tuned the transmitted beam by using an asymmetric-lattice metal nanohole. These works demonstrate tunability the visible spectrum, whereas the target frequency of most tuned MMs is within the NIR or MIR.

## 6. Conclusions

This paper reviews methods tune MM reversibly within a certain range. We considered the effects of thermal, optical, mechanical, and electrical factors. When a particular material or structure in an MM is excited by heat, light, mechanical force, and voltage, the property of MM can change reversibly. VO_2_ and GST are phase-change materials and representative components of thermally-tunable MM. ITO as a carrier adjustable material is widely used in optically and electrically tunable MMs. PDMS is widely used in MMs that are mechanically tuned by stretchable substrates. This review expresses the working principles or mechanisms of the classified tunable MMs. Research has manipulated LSPR, absorption, transmittance, reflectance, polarization, and thermal emission.

Each method has the advantages and disadvantages of visible tunability, tuning range, and modulation speed. A strength of one method can be a weakness of other methods, so the choice of an appropriate tuning method is dictated by the purpose of tuning. Research on tunable MMs has achieved many advances, such as by increasing the range of variation in MMs or using novel materials. Further developments of this technology will yield practical applications of MMs.
